# 4-[(5-Bromo-2-hydroxy­benzyl­idene)amino]-*N*-(4,6-dimethyl­pyrimidin-2-yl)benzene­sulfonamide–4-bromo-2-[(*E*)-({4-[(4,6-dimethyl­pyrimidin-2-yl)sulfamo­yl]phen­yl}iminio)meth­yl]phenolate [0.61 (7)/0.39 (7)]

**DOI:** 10.1107/S1600536808041214

**Published:** 2008-12-13

**Authors:** Hazoor A. Shad, M. Nawaz Tahir, Zahid H. Chohan

**Affiliations:** a Department of Chemistry, Bahauddin Zakariya University, Multan-60800, Pakistan; b University of Sargodha, Department of Physics, Sargodha, Pakistan

## Abstract

The title compound, 0.61C_19_H_17_BrN_4_O_3_S·0.39C_19_H_17_BrN_4_O_3_S, is a Schiff base derived from 5-bromo­salicylaldehyde and 4-amino-*N*-(4,6-dimethyl-2-pyrimidin­yl)benzene­sulfonamide(sulfamethazine) and is isostructural with its chloro analogue. The geometry of the title mol­ecule points to the enol (OH—C=C—C=N) form as the major tautomer, however two electron-density maxima corresponding to the H atoms of the OH and NH groups, found in the region of a strong intra­molecular N⋯H⋯O hydrogen bond, do not allow the elimination of the presence of the zwitterionic (O^−^—C=C—C=NH^+^) form in the crystal. Refinement of the occupancies of these H atoms gave a 0.61 (7):0.39 (7) ratio of the enolic and zwitterionic forms. The two benzene rings within the mol­ecule are nearly coplanar and the central benzene ring forms a dihedral angle of 84.1 (1)° with the pyrimidine fragment. An inter­molecular N—H⋯O hydrogen bond links mol­ecules into chains extended along the *a* axis and a C—H⋯O link is also present. The H atoms of one of the methyl groups are disordered over two sites with an occupancy ratio of 0.72 (7):0.28 (7).

## Related literature

For the crystal structures of similar sulphonamides, see: Chohan *et al.* (2008*a* ▶,*b* ▶); Shad *et al.* (2008 ▶); Tahir *et al.* (2008 ▶). 
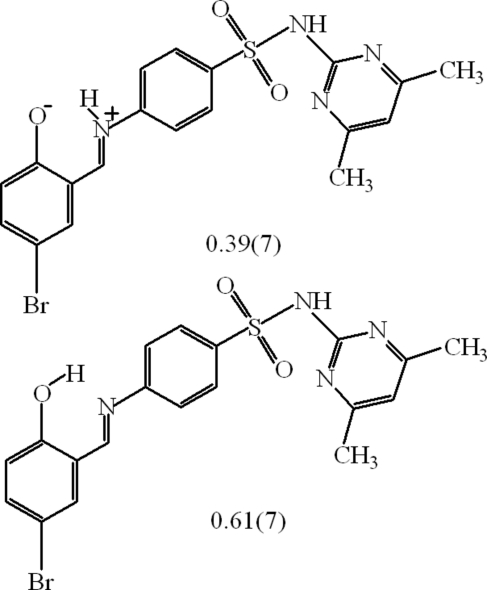


## Experimental

### 

#### Crystal data

0.61C_19_H_17_BrN_4_O_3_S·0.39C_19_H_17_BrN_4_O_3_S
*M* *_r_* = 461.34
Orthorhombic, 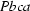

*a* = 11.7919 (9) Å
*b* = 13.9965 (8) Å
*c* = 23.5117 (17) Å
*V* = 3880.5 (5) Å^3^
*Z* = 8
Mo *K*α radiation
μ = 2.26 mm^−1^
*T* = 296 (2) K
0.20 × 0.16 × 0.14 mm

#### Data collection

Bruker KAPPA APEXII CCD diffractometer
Absorption correction: multi-scan (*SADABS*; Bruker, 2005 ▶) *T*_min_ = 0.650, *T*_max_ = 0.725
19597 measured reflections
3428 independent reflections
1961 reflections with *I* > 2σ(*I*)
*R*_int_ = 0.081

#### Refinement

*R*[*F* ^2^ > 2σ(*F* ^2^)] = 0.048
*wR*(*F* ^2^) = 0.131
*S* = 1.00
3428 reflections
257 parameters
H-atom parameters constrained
Δρ_max_ = 0.35 e Å^−3^
Δρ_min_ = −0.65 e Å^−3^

### 

Data collection: *APEX2* (Bruker, 2007 ▶); cell refinement: *SAINT* (Bruker, 2007 ▶); data reduction: *SAINT*; program(s) used to solve structure: *SHELXS97* (Sheldrick, 2008 ▶); program(s) used to refine structure: *SHELXL97* (Sheldrick, 2008 ▶); molecular graphics: *ORTEP-3 for Windows* (Farrugia, 1997 ▶) and *PLATON* (Spek, 2003 ▶); software used to prepare material for publication: *WinGX* publication routines (Farrugia, 1999 ▶) and *PLATON*.

## Supplementary Material

Crystal structure: contains datablocks global, I. DOI: 10.1107/S1600536808041214/gk2160sup1.cif

Structure factors: contains datablocks I. DOI: 10.1107/S1600536808041214/gk2160Isup2.hkl

Additional supplementary materials:  crystallographic information; 3D view; checkCIF report

## Figures and Tables

**Table 1:** Hydrogen-bond geometry (Å, °)

*D*—H⋯*A*	*D*—H	H⋯*A*	*D*⋯*A*	*D*—H⋯*A*
N1—H1*N*⋯O1	1.06	1.73	2.530 (5)	129
O1—H1*O*⋯N1	0.86	1.94	2.530 (5)	124
N2—H2*N*⋯O1^i^	0.86	2.20	2.871 (4)	135
C9—H9⋯O2^ii^	0.93	2.50	3.417 (5)	169

Symmetry codes: (i) 
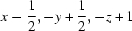
; (ii) 
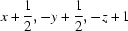
.

**Fig. 1.**
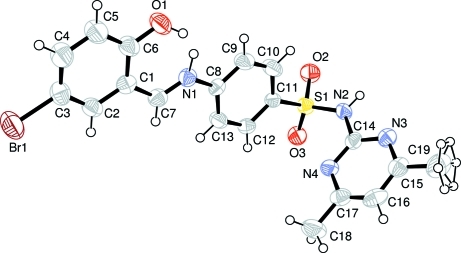
Molecular structure of the title compound, with the atom numbering scheme. The thermal ellipsoids are drawn at the 30% probability level. H-atoms are shown by small circles of arbitrary radii. The H atoms bonded to O1 and N1 show partial occupancy.

**Fig. 2.**
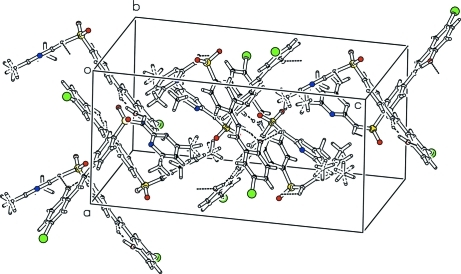
Crystal packing of the title compound.

## supplementary crystallographic information

### Comment

As a result of vital pharmacological effects of sulfonamide and their derivatives, there is a rising attention in synthesizing and biotesting of these derivatives. In the vision of the versatile biological chemistry of sulfonamides, we have synthesized and recently published the crystal structures of several compounds from this group (Chohan *et al.*, 2008*a*, 2008*b;* Shad *et al.*, 2008; Tahir *et al.*, 2008). In the same continuation, we herein report the structure of the title compound.

The title compound (I) (Fig. 1) was prepared from sulfamethazine and 5-bromosalicylaldehyde. The crystal of the title compound is isostructural with 4-(5-chloro-2-hydroxybenzylideneamino)-*N*-(4,6-dimethylpyrimidin-2-yl)benzenesulfonamide (II) (Chohan *et al.*, 2008*b*). In the crystal two tautomers,  enolic and zwitterionic, with an approximate ratio of 3:2 coexists, as shown by the refinement of H atom occupancies from the N-H and O-H groups.

We shall restrict discussion to comparison of the bond geometry between (I) and (II). The longest bond in the molecule is C3—Br1, having bond distance 1.890 (5) Å. The bond distances in (I), S1—N2 [1.629 (4) Å] and S1—C11 [1.753 (4) Å] remain equal within experimental errors with those observed in (II). The range of S—O [1.416 (3)–1.431 (3) Å] bond lengths is increased compared to 1.422 (2)–1.4282 (19) Å in (II). The bond angles around the S1-atom are slightly changed. The geometry of intramolecular as well as intermolecular H-bonding is given in Table 1 and shown in Fig 2.

### Experimental

Sulfamethazine (0.5566 g, 2 mmol) in ethanol (15 ml) was reacted with ethanolic (10 ml) solution of 5-bromosalicylaldehyde (0.4020 g, 2 mmol). The mixture was refluxed for 3 h. The colour of the solution gradually changed from colourless to orange-red. The solution was then cooled to room temperature, filtered and volume reduced to about one-third on rotary evaporator. After 12 days crystals of the title compound were obtained.

### Refinement

The positions of H-atoms attached to O1 and N1 were determined from the difference Fourier synthesis and in the refinement these atoms were constrained to ride on their parent atoms. Their occupancy factors were allowed to refine with the the sum of the occupancy factors constrained to 1.00. Remaining H-atoms were positioned geometrically, with C—H = 0.93-0.96 Å. The *U*_iso_(H) = *xU*_eq_(C, N, O), where *x* = 1.5 for methyl H, H1N, H1O and *x* = 1.2 for all other H atoms. The H-atoms of one of the methyl groups are disordered over two sites with occupancy ratio of 72:28.

### Crystal data

**Table:**

0.61C_19_H_17_BrN_4_O_3_S·0.39C_19_H_17_BrN_4_O_3_S	*D*_x_ = 1.579 Mg m^−^^3^
*M**_r_* = 461.34	Melting point: 497 K
Orthorhombic, *P**b**c**a*	Mo *K*α radiation, λ = 0.71073 Å
Hall symbol: -P 2ac 2ab	Cell parameters from 3428 reflections
*a* = 11.7919 (9) Å	θ = 2.4–25.0°
*b* = 13.9965 (8) Å	µ = 2.26 mm^−^^1^
*c* = 23.5117 (17) Å	*T* = 296 K
*V* = 3880.5 (5) Å^3^	Prismatic, red
*Z* = 8	0.20 × 0.16 × 0.14 mm
*F*(000) = 1872	

### Data collection

**Table:**

Bruker KAPPA APEXII CCD diffractometer	3428 independent reflections
Radiation source: fine-focus sealed tube	1961 reflections with *I* > 2σ(*I*)
graphite	*R*_int_ = 0.081
Detector resolution: 7.9 pixels mm^-1^	θ_max_ = 25.0°, θ_min_ = 2.4°
ω scans	*h* = −14→14
Absorption correction: multi-scan (*SADABS*; Bruker, 2005)	*k* = −16→16
*T*_min_ = 0.650, *T*_max_ = 0.725	*l* = −27→26
19597 measured reflections	

### Refinement

**Table:**

Refinement on *F*^2^	Primary atom site location: structure-invariant direct methods
Least-squares matrix: full	Secondary atom site location: difference Fourier map
*R*[*F*^2^ > 2σ(*F*^2^)] = 0.048	Hydrogen site location: inferred from neighbouring sites
*wR*(*F*^2^) = 0.131	H-atom parameters constrained
*S* = 1.00	*w* = 1/[σ^2^(*F*_o_^2^) + (0.057*P*)^2^ + 2.1678*P*] where *P* = (*F*_o_^2^ + 2*F*_c_^2^)/3
3428 reflections	(Δ/σ)_max_ = 0.001
257 parameters	Δρ_max_ = 0.35 e Å^−^^3^
0 restraints	Δρ_min_ = −0.65 e Å^−^^3^

### Special details

**Table:**

Geometry. All e.s.d.'s (except the e.s.d. in the dihedral angle between two l.s. planes) are estimated using the full covariance matrix. The cell e.s.d.'s are taken into account individually in the estimation of e.s.d.'s in distances, angles and torsion angles; correlations between e.s.d.'s in cell parameters are only used when they are defined by crystal symmetry. An approximate (isotropic) treatment of cell e.s.d.'s is used for estimating e.s.d.'s involving l.s. planes.
Refinement. Refinement of *F*^2^ against ALL reflections. The weighted *R*-factor *wR* and goodness of fit *S* are based on *F*^2^, conventional *R*-factors *R* are based on *F*, with *F* set to zero for negative *F*^2^. The threshold expression of *F*^2^ > σ(*F*^2^) is used only for calculating *R*-factors(gt) *etc*. and is not relevant to the choice of reflections for refinement. *R*-factors based on *F*^2^ are statistically about twice as large as those based on *F*, and *R*- factors based on ALL data will be even larger.

### Fractional atomic coordinates and isotropic or equivalent isotropic displacement parameters (Å^2^)

**Table:**

	*x*	*y*	*z*	*U*_iso_*/*U*_eq_	Occ. (<1)
Br1	1.18229 (5)	0.76268 (4)	0.36991 (3)	0.0824 (3)	
S1	0.40502 (9)	0.37094 (8)	0.60341 (5)	0.0417 (3)	
O1	0.9535 (3)	0.3837 (2)	0.39965 (16)	0.0716 (11)	
H1O	0.9124	0.3692	0.4287	0.086*	0.61 (7)
N1	0.8172 (3)	0.4593 (3)	0.46953 (16)	0.0426 (9)	
H1N	0.8601	0.3968	0.4558	0.051*	0.39 (7)
O2	0.3377 (3)	0.3110 (2)	0.56749 (13)	0.0560 (9)	
O3	0.3565 (2)	0.4558 (2)	0.62541 (13)	0.0511 (8)	
N2	0.4430 (3)	0.2997 (2)	0.65478 (15)	0.0452 (10)	
H2N	0.4177	0.2421	0.6535	0.068*	
N3	0.5374 (3)	0.2497 (3)	0.73414 (17)	0.0472 (10)	
N4	0.5447 (3)	0.4138 (2)	0.70529 (16)	0.0458 (9)	
C1	0.9643 (4)	0.5482 (3)	0.42496 (19)	0.0440 (11)	
C2	1.0200 (4)	0.6363 (3)	0.4176 (2)	0.0493 (12)	
H2	0.9966	0.6894	0.4383	0.059*	
C3	1.1079 (4)	0.6443 (3)	0.3803 (2)	0.0538 (13)	
C4	1.1447 (4)	0.5657 (4)	0.3499 (2)	0.0661 (15)	
H4	1.2054	0.5716	0.3249	0.079*	
C5	1.0922 (4)	0.4790 (4)	0.3565 (2)	0.0685 (16)	
H5	1.1168	0.4269	0.3352	0.082*	
C6	1.0036 (4)	0.4677 (3)	0.3942 (2)	0.0532 (13)	
C7	0.8679 (4)	0.5393 (3)	0.46274 (19)	0.0450 (11)	
H7	0.8424	0.5927	0.4824	0.054*	
C8	0.7198 (3)	0.4440 (3)	0.50347 (18)	0.0375 (10)	
C9	0.6759 (4)	0.3538 (3)	0.5027 (2)	0.0538 (13)	
H9	0.7107	0.3067	0.4809	0.065*	
C10	0.5808 (4)	0.3317 (3)	0.5338 (2)	0.0548 (13)	
H10	0.5514	0.2701	0.5329	0.066*	
C11	0.5290 (3)	0.4005 (3)	0.56628 (17)	0.0359 (10)	
C12	0.5738 (4)	0.4917 (3)	0.56843 (19)	0.0430 (11)	
H12	0.5405	0.5382	0.5912	0.052*	
C13	0.6683 (4)	0.5127 (3)	0.5365 (2)	0.0457 (12)	
H13	0.6979	0.5743	0.5372	0.055*	
C14	0.5124 (3)	0.3231 (3)	0.70054 (19)	0.0417 (11)	
C15	0.6020 (4)	0.2704 (3)	0.7790 (2)	0.0523 (13)	
C16	0.6396 (4)	0.3624 (4)	0.7881 (2)	0.0579 (13)	
H16	0.6850	0.3764	0.8193	0.069*	
C17	0.6095 (4)	0.4328 (3)	0.7507 (2)	0.0501 (12)	
C18	0.6482 (5)	0.5342 (4)	0.7575 (3)	0.0784 (17)	
H18A	0.6846	0.5550	0.7231	0.118*	
H18B	0.7009	0.5382	0.7885	0.118*	
H18C	0.5840	0.5743	0.7652	0.118*	
C19	0.6307 (5)	0.1897 (4)	0.8188 (2)	0.0797 (18)	
H19A	0.5973	0.1316	0.8050	0.120*	0.72 (7)
H19B	0.6015	0.2036	0.8560	0.120*	0.72 (7)
H19C	0.7115	0.1824	0.8208	0.120*	0.72 (7)
H19D	0.6762	0.2134	0.8495	0.120*	0.28 (7)
H19E	0.6720	0.1414	0.7985	0.120*	0.28 (7)
H19F	0.5620	0.1626	0.8337	0.120*	0.28 (7)

### Atomic displacement parameters (Å^2^)

**Table:**

	*U*^11^	*U*^22^	*U*^33^	*U*^12^	*U*^13^	*U*^23^
Br1	0.0841 (5)	0.0587 (4)	0.1044 (6)	−0.0198 (3)	0.0112 (4)	0.0058 (3)
S1	0.0396 (6)	0.0417 (6)	0.0437 (7)	−0.0038 (5)	−0.0033 (5)	0.0005 (6)
O1	0.086 (3)	0.0396 (19)	0.089 (3)	−0.0101 (18)	0.039 (2)	−0.0107 (19)
N1	0.044 (2)	0.043 (2)	0.041 (2)	0.0041 (18)	0.0030 (19)	0.0006 (18)
O2	0.053 (2)	0.060 (2)	0.056 (2)	−0.0146 (16)	−0.0152 (17)	−0.0017 (17)
O3	0.0480 (18)	0.0495 (19)	0.056 (2)	0.0038 (15)	0.0073 (16)	−0.0015 (16)
N2	0.055 (2)	0.039 (2)	0.042 (2)	−0.0141 (18)	−0.010 (2)	0.0017 (18)
N3	0.047 (2)	0.051 (2)	0.043 (3)	0.0018 (18)	−0.003 (2)	0.000 (2)
N4	0.050 (2)	0.046 (2)	0.042 (3)	−0.0097 (18)	0.001 (2)	−0.0051 (19)
C1	0.042 (3)	0.049 (3)	0.041 (3)	0.005 (2)	−0.006 (2)	0.000 (2)
C2	0.056 (3)	0.038 (3)	0.054 (3)	0.002 (2)	−0.003 (3)	−0.003 (2)
C3	0.043 (3)	0.052 (3)	0.066 (4)	−0.003 (2)	0.002 (3)	0.006 (3)
C4	0.053 (3)	0.060 (3)	0.086 (4)	0.002 (3)	0.028 (3)	−0.002 (3)
C5	0.063 (3)	0.052 (3)	0.091 (5)	0.001 (3)	0.031 (3)	−0.010 (3)
C6	0.056 (3)	0.041 (3)	0.063 (4)	0.003 (2)	0.008 (3)	−0.003 (3)
C7	0.051 (3)	0.041 (3)	0.044 (3)	0.010 (2)	0.000 (2)	−0.002 (2)
C8	0.037 (2)	0.043 (3)	0.033 (3)	0.004 (2)	0.002 (2)	0.001 (2)
C9	0.060 (3)	0.044 (3)	0.058 (3)	0.002 (2)	0.016 (3)	−0.013 (2)
C10	0.059 (3)	0.038 (3)	0.067 (4)	−0.006 (2)	0.011 (3)	−0.011 (2)
C11	0.040 (2)	0.034 (2)	0.034 (3)	0.0019 (19)	−0.004 (2)	−0.0028 (19)
C12	0.050 (3)	0.039 (3)	0.040 (3)	0.005 (2)	0.008 (2)	−0.007 (2)
C13	0.050 (3)	0.035 (2)	0.052 (3)	−0.004 (2)	0.006 (2)	−0.006 (2)
C14	0.041 (3)	0.048 (3)	0.036 (3)	−0.002 (2)	0.004 (2)	−0.001 (2)
C15	0.054 (3)	0.057 (3)	0.046 (3)	0.016 (3)	0.007 (3)	−0.003 (3)
C16	0.046 (3)	0.082 (4)	0.046 (3)	0.008 (3)	−0.009 (2)	−0.016 (3)
C17	0.043 (3)	0.061 (3)	0.046 (3)	−0.005 (2)	0.005 (2)	−0.012 (3)
C18	0.085 (4)	0.070 (4)	0.081 (5)	−0.031 (3)	−0.002 (3)	−0.020 (3)
C19	0.095 (4)	0.083 (4)	0.061 (4)	0.035 (3)	−0.016 (3)	0.002 (3)

### Geometric parameters (Å, °)

**Table:**

Br1—C3	1.890 (5)	C7—H7	0.9300
S1—O3	1.416 (3)	C8—C9	1.365 (6)
S1—O2	1.431 (3)	C8—C13	1.378 (6)
S1—N2	1.629 (4)	C9—C10	1.374 (6)
S1—C11	1.753 (4)	C9—H9	0.9300
O1—C6	1.322 (5)	C10—C11	1.372 (6)
O1—H1O	0.8621	C10—H10	0.9300
N1—C7	1.279 (5)	C11—C12	1.381 (5)
N1—C8	1.414 (5)	C12—C13	1.375 (6)
N1—H1N	1.0611	C12—H12	0.9300
N2—C14	1.391 (5)	C13—H13	0.9300
N2—H2N	0.8600	C15—C16	1.378 (6)
N3—C14	1.329 (5)	C15—C19	1.506 (7)
N3—C15	1.332 (6)	C16—C17	1.368 (6)
N4—C14	1.330 (5)	C16—H16	0.9300
N4—C17	1.339 (6)	C17—C18	1.499 (6)
C1—C2	1.408 (6)	C18—H18A	0.9600
C1—C6	1.416 (6)	C18—H18B	0.9600
C1—C7	1.448 (6)	C18—H18C	0.9600
C2—C3	1.362 (6)	C19—H19A	0.9600
C2—H2	0.9300	C19—H19B	0.9600
C3—C4	1.382 (7)	C19—H19C	0.9600
C4—C5	1.371 (6)	C19—H19D	0.9600
C4—H4	0.9300	C19—H19E	0.9600
C5—C6	1.380 (7)	C19—H19F	0.9600
C5—H5	0.9300		
			
O3—S1—O2	118.9 (2)	C11—C10—C9	120.1 (4)
O3—S1—N2	110.7 (2)	C11—C10—H10	120.0
O2—S1—N2	103.37 (18)	C9—C10—H10	120.0
O3—S1—C11	108.73 (19)	C10—C11—C12	119.9 (4)
O2—S1—C11	107.90 (19)	C10—C11—S1	118.8 (3)
N2—S1—C11	106.54 (18)	C12—C11—S1	121.3 (3)
C6—O1—H1O	122.5	C13—C12—C11	119.2 (4)
C7—N1—C8	125.7 (4)	C13—C12—H12	120.4
C7—N1—H1N	117.4	C11—C12—H12	120.4
C8—N1—H1N	115.7	C12—C13—C8	121.0 (4)
C14—N2—S1	126.3 (3)	C12—C13—H13	119.5
C14—N2—H2N	116.9	C8—C13—H13	119.5
S1—N2—H2N	116.9	N3—C14—N4	128.6 (4)
C14—N3—C15	115.4 (4)	N3—C14—N2	114.1 (4)
C14—N4—C17	114.8 (4)	N4—C14—N2	117.2 (4)
C2—C1—C6	118.8 (4)	N3—C15—C16	120.7 (5)
C2—C1—C7	121.1 (4)	N3—C15—C19	117.2 (5)
C6—C1—C7	120.1 (4)	C16—C15—C19	122.2 (5)
C3—C2—C1	120.4 (4)	C17—C16—C15	119.3 (5)
C3—C2—H2	119.8	C17—C16—H16	120.3
C1—C2—H2	119.8	C15—C16—H16	120.3
C2—C3—C4	120.4 (4)	N4—C17—C16	121.1 (4)
C2—C3—Br1	120.6 (4)	N4—C17—C18	116.6 (5)
C4—C3—Br1	119.0 (4)	C16—C17—C18	122.3 (5)
C5—C4—C3	120.3 (5)	C17—C18—H18A	109.5
C5—C4—H4	119.8	C17—C18—H18B	109.5
C3—C4—H4	119.8	H18A—C18—H18B	109.5
C4—C5—C6	121.1 (5)	C17—C18—H18C	109.5
C4—C5—H5	119.5	H18A—C18—H18C	109.5
C6—C5—H5	119.5	H18B—C18—H18C	109.5
O1—C6—C5	120.2 (4)	C15—C19—H19A	109.5
O1—C6—C1	120.8 (4)	C15—C19—H19B	109.5
C5—C6—C1	119.0 (4)	H19A—C19—H19B	109.5
N1—C7—C1	121.3 (4)	C15—C19—H19C	109.5
N1—C7—H7	119.4	H19A—C19—H19C	109.5
C1—C7—H7	119.4	H19B—C19—H19C	109.5
C9—C8—C13	119.1 (4)	C15—C19—H19D	109.5
C9—C8—N1	116.2 (4)	C15—C19—H19E	109.5
C13—C8—N1	124.7 (4)	H19D—C19—H19E	109.5
C8—C9—C10	120.7 (4)	C15—C19—H19F	109.5
C8—C9—H9	119.6	H19D—C19—H19F	109.5
C10—C9—H9	119.6	H19E—C19—H19F	109.5
			
O3—S1—N2—C14	−53.3 (4)	O3—S1—C11—C10	−169.9 (4)
O2—S1—N2—C14	178.3 (4)	O2—S1—C11—C10	−39.7 (4)
C11—S1—N2—C14	64.7 (4)	N2—S1—C11—C10	70.8 (4)
C6—C1—C2—C3	1.9 (7)	O3—S1—C11—C12	9.0 (4)
C7—C1—C2—C3	−177.5 (4)	O2—S1—C11—C12	139.2 (3)
C1—C2—C3—C4	−1.1 (7)	N2—S1—C11—C12	−110.4 (4)
C1—C2—C3—Br1	179.6 (3)	C10—C11—C12—C13	1.9 (7)
C2—C3—C4—C5	0.8 (8)	S1—C11—C12—C13	−177.0 (3)
Br1—C3—C4—C5	−179.9 (4)	C11—C12—C13—C8	−1.3 (7)
C3—C4—C5—C6	−1.4 (9)	C9—C8—C13—C12	−0.1 (7)
C4—C5—C6—O1	179.4 (5)	N1—C8—C13—C12	−179.8 (4)
C4—C5—C6—C1	2.2 (8)	C15—N3—C14—N4	1.1 (7)
C2—C1—C6—O1	−179.6 (4)	C15—N3—C14—N2	−178.7 (4)
C7—C1—C6—O1	−0.2 (7)	C17—N4—C14—N3	−1.1 (7)
C2—C1—C6—C5	−2.4 (7)	C17—N4—C14—N2	178.7 (4)
C7—C1—C6—C5	177.0 (5)	S1—N2—C14—N3	−175.0 (3)
C8—N1—C7—C1	−177.3 (4)	S1—N2—C14—N4	5.2 (6)
C2—C1—C7—N1	180.0 (4)	C14—N3—C15—C16	−0.7 (6)
C6—C1—C7—N1	0.6 (7)	C14—N3—C15—C19	179.0 (4)
C7—N1—C8—C9	177.0 (4)	N3—C15—C16—C17	0.4 (7)
C7—N1—C8—C13	−3.2 (7)	C19—C15—C16—C17	−179.3 (5)
C13—C8—C9—C10	0.8 (7)	C14—N4—C17—C16	0.7 (6)
N1—C8—C9—C10	−179.4 (4)	C14—N4—C17—C18	179.7 (4)
C8—C9—C10—C11	−0.3 (7)	C15—C16—C17—N4	−0.4 (7)
C9—C10—C11—C12	−1.1 (7)	C15—C16—C17—C18	−179.4 (5)
C9—C10—C11—S1	177.8 (4)		

### Hydrogen-bond geometry (Å, °)

**Table:**

*D*—H···*A*	*D*—H	H···*A*	*D*···*A*	*D*—H···*A*
N1—H1N···O1	1.06	1.73	2.530 (5)	129
O1—H1O···N1	0.86	1.94	2.530 (5)	124
N2—H2N···O1^i^	0.86	2.20	2.871 (4)	135
C9—H9···O2^ii^	0.93	2.50	3.417 (5)	169

Symmetry codes: (i) *x*−1/2, −*y*+1/2, −*z*+1; (ii) *x*+1/2, −*y*+1/2, −*z*+1.

## References

[bb1] Bruker (2005). *SADABS* Bruker AXS Inc. Madison, Wisconsin, USA.

[bb2] Bruker (2007). *APEX2* and *SAINT* . Bruker AXS Inc. Madison, Wisconsin, USA.

[bb3] Chohan, Z. H., Shad, H. A., Tahir, M. N. & Khan, I. U. (2008*a*). *Acta Cryst.* E**64**, o725. 10.1107/S1600536808005084 PMC2960939 21202115

[bb4] Chohan, Z. H., Tahir, M. N., Shad, H. A. & Khan, I. U. (2008*b*). *Acta Cryst.* E**64**, o648. 10.1107/S1600536808005606 PMC2960946 21202046

[bb5] Farrugia, L. J. (1997). *J. Appl. Cryst.* **30**, 565.

[bb6] Farrugia, L. J. (1999). *J. Appl. Cryst.* **32**, 837–838.

[bb7] Shad, H. A., Chohan, Z. H., Tahir, M. N. & Khan, I. U. (2008). *Acta Cryst.* E**64**, o635. 10.1107/S1600536808005321 PMC2960758 21201966

[bb8] Sheldrick, G. M. (2008). *Acta Cryst.* A**64**, 112–122. 10.1107/S0108767307043930 18156677

[bb9] Spek, A. L. (2003). *J. Appl. Cryst.* **36**, 7–13.

[bb10] Tahir, M. N., Chohan, Z. H., Shad, H. A. & Khan, I. U. (2008). *Acta Cryst.* E**64**, o720. 10.1107/S160053680800682X PMC2960962 21202110

